# Our Experience with Kashimas Procedure for Bilateral Abductor Vocal Cord Palsy 

**DOI:** 10.22038/ijorl.2019.33863.2126

**Published:** 2020-09

**Authors:** Tabish Maqbool, Rauf Ahmed, Ihsan Ali

**Affiliations:** 1 *Department of Otorhinolaryngology, Head and Neck Surgery,* *Govt. Medical College Srinagar, Srinagar, India. *

**Keywords:** Bilateral vocal cord paralysis, Endoscopic cordotomy, Voice

## Abstract

**Introduction:**

Kashima operation, also known as endoscopic laser cordotomy is used for the treatment of bilateral abductor vocal cord palsy where the glottis chink is made posteriorly, sufficient enough for patient to breathe comfortably without any strider.

**Materials and Methods:**

This Clinical Trial Was Performed On 12 Patients with Bilateral Abductor Vocal Cord Paralysis. All Patients Underwent Kashimas Procedure and Post-Operative Voice, Respiratory and Deglutition Function Were Evaluated.

**Results:**

75% of patients were females and the mean age of patients was 40.9 ±9.13 years. In our patients, the most common etiology of bilateral vocal cord palsy was thyroid surgery (n=10, 83.33%).There was a significant improvement in breathing after surgery (P=0.001). After the procedure, 70% of patient had mild voice handicap score, and MPT was in normal range in 91.6% of cases.

**Conclusion:**

Kashimas procedure is a satisfactory surgical treatment for treating bilateral vocal cord palsy in regards to strider. No aspiration was seen in any of the patients post-surgery and voice outcome of these patients was also satisfactory.

## Introduction

Vocal cord palsy can be unilateral or bilateral. Unilateral vocal cord palsy is usually compensated by the movement of opposite cord while in case of bilateral palsy the vocal cords can remain abducted (in adductor palsy) which can lead to aspiration pneumonia or can remain in midline (abductor palsy) leading to airway compromise. Bilateral immobile vocal cord due to bilateral abductor palsy leads to respiratory distress that occasionally can become life threatening. Vocal cords are immobilized either due to palsy or fixation.In abductor vocal cord palsy the voice quality of patient remains fine but, the patient does have stridor which gets worse on exertion or during respiratory infection.  Unilateral vocal fold paralysis is seen to be more common; however the exact incidence of bilateral abductor vocal cord palsy is unknown in the current literature (1).  

Most common causes of bilateral abductor vocal cord palsy include surgical trauma (44%), malignancies (17%), endotracheal intubation (15%), while neurologic causes and idiopathic cases account for 12% each (2). The most common cause remains iatrogenic injury during thyroidectomy (3). Primary objective of the treatment of BVFP is to improve the ventilation of patients. Both the external and endoscopic methods can be used in improving the stenotic airway. Initially tracheostomy was used as the main treatment method. Various surgical techniques from simple ventriculocordectomy (by Chevalier Jackson in 1922) to complex procedures like reanimation of the larynx with an electrical device have been described till date (4,5). Other techniques which were used is arytenoidectomy, in which the removal of arytenoids cartilage leads to expansion of glottal inlet. 

It was initially done externally and then endoscopic technique of the same was introduced. Another technique is Laterofixation of the vocal cord and/or arytenoids cartilage, in which, after the proper exposure of larynx, thyroarytenoid muscle is removed and sutures are introduced in to the larynx from outside through 2 needles. Finally the sutures are pulled through the laryngoscope which helps in lateralizing the cord. Newer methods which are under investigation are reinnervation procedures like RLN anastomosis (phrenic nerve as a source) and transplantation of muscle nerve pedicle from omohyoid or sternohyoid to the posterior cricoarytenoid muscle procedures for reinnervating laryngeal muscle and Functional electrical stimulation (FES) of paralyzed laryngeal muscles. Botox injection into bilateral adductor muscles has also been described by Marie et al .Various techniques of endoscopic approach have also been proposed and modified by various surgeons (6,7). 

Remacle et al., and Plouin-Gaudon et al., did subtotal arytenoidectomy using C02 laser (8,9).

Oswal et al., used the parameters of respiration, phonation and swallowing to describe their results of endoscopic laser surgery for bilateral Vocal cord palsy (10). CO _2_ laser has an advantage having increased precision, better hemostasis and minimal tissue handling. In 1989, Kashima introduced the most successful laterization procedure, endoscopic laser posterior cordotomy, in which vocal fold is cut transversely just in front of the vocal process. Tissue resection advances laterally until the inner perichondrium of thyroid and cricoids cartilage is reached (11). This prospective study was conducted in the department of Otorhinolaryngology and HNS, from November 2015 to February 2018. Twelve patients who presented with bilateral abductor vocal cord palsy and ultimately underwent CO2 laser posterior cordotomy as the surgical procedure were included in the study.

## Materials and Methods

This prospective study was conducted in our department which is a tertiary care referral center, from November 2015 to February 2018. This study aimed to check the postoperative effect of Kashimas procedure on: 1. voice, depending on VHI, MPT and GRBAS score, 2.swallowing, depending upon the complaints of aspiration by patient postoperatively, and 3. respiration by comparing preoperative and postoperative dyspnea index score, which is a subjective measure of breathing quality. Twelve patients who presented to our OPD with Bilateral abductor vocal cord palsy and ultimately underwent CO_2_ laser posterior cordotomy as the surgical procedure were included in the study. All patients gave written informed consent before the procedure. A detailed history and clinical examination was taken. The surgical procedure was performed under general anesthesia. Cuffed endotracheal laser tube was used. Neurosurgical patties moistened with saline and attached to long threads which were kept outside the cope were placed in subglottis to protect the cuffed tube from thermal damage. We used the CO_2_ laser with micromanipulator in a repeat super pulse mode and power of 7 W. Suction tip was used to identify the vocal process of arytenoid cartilage.

The part of vocal cord just anterior to it was vaporized transversely; the entire vocalis and thyroarytenoid muscle was vaporized avoiding injury to the vocal process. 

Tissue resection was advanced laterally until the inner perichondrium of thyroid was reached. Contraction and retraction of the vocalis muscle would lead to a wedge-shaped defect in the posterior cord, thus, improving the glottic chink to about 5–6 mm.

Patients were followed-up regularly for a minimum period of 6 months. Surgical outcome was assessed by following measures: effect of Kashimas procedure on voice, depending on VHI (0-30-mild minimal amount of handicap, 31-60- moderate, 60-120-severe), MPT and GRBAS score, swallowing, depending upon the complaints of aspiration by patient postoperatively, and respiration by comparing preoperative and postoperative dyspnea index score, which is a subjective measure of breathing quality. All patients included in the study were having mild VHI, and MPT >10.

## Results

This study was prospective in design and included 12 patients. The mean age of the patients was 40.9 ± 9.1.

The most common etiology was thyroid surgery (n=10, 83.33%) while in 16.66% of patients (n=2) cause of vocal cord palsy was idiopathic. 

Effect on phonation was studied on basis of VHI, maximum phonation time (MPT) and GRBAS score. VHI values demonstrated that while nine patients (75%) had no/mild degree of voice handicap, two patients (16.6%) had moderate and one patient (8.33%) had a severe degree of handicap. While 11 patients (91.6%) had a normal MPT (above 10), one patients (8.33%) had a reduced MPT (10 or below).

The difference in the preoperative and postoperative mean was 9.3 based on dyspnea index score and the p value was 0.001 which is statistically significant.

None of the patients in our study complained of aspiration postoperatively.

## Discussion

The main aim in treating bilateral vocal cord paralysis is to allow the patient to breathe normally with maintenance of Swallowing and phonation. Spontaneous recovery occurs in cases where vocal cord palsy is a result of axonotmesis, where neuroregeneration occurs at a rate o t f 1 to 3 mm/day (12).

In our study majority of the patients were females (75%) and the major etiology was seen to be thyroid surgery (83.3%).

In a study done by Francesco Dispenza et al he described in his study that 73.3% of patients were females and thyroid surgery was the etiology in 100% of the patients (13). This study is thus in accordance with our study. Hans Edmund Eckel in his study found showed Subclinical aspiration occurred in 5 of 10 patients after he did arytenoidectomy (14), but in none of 18 patients after cordectomy.So aspiration is a problem with arytenoidectomy which does not occur in cordectomy. Raghavendra rao in his study of 25 patients divided them into 2 groups (15). Group 1 underwent vocal cord lateralization (15pts) while group 2 (10 patients) underwent posterior cordotomy. They saw that 100% of patients in group 2 had grade 0 postoperative airway compared to 76.9% in group 1.80% of group 2 patients had good postoperative subjective voice quality compared to only 23%of group 1 patients. These studies showed that arytenoidectomy and VC lateralization are not superior to posterior cordotomy. According to a study done by Nitish Virmani where he studied 7 patients and found   that four patients (57.1%) had no/mild degree of voice handicap, two patients (28.5%) had moderate and one patient (14.28%) had a severe degree of handicap according to VHI score (16). 

While five patients (66.7%) had a normal MPT, two patients (33.3%) had a reduced MPT). In our study Dyspnea index score (Table 1) was used to see the surgical outcome for effect on respiration. Also our (Table.2). 

**Table 1:** Dyspnea index score

**Dyspnea at rest**	**0** ** (nil)**	**1** **(mild)**	**2 (moderate)**	**3 (severe)**
Dyspnea at light work	0	1	2	3
Dyspnea at exertion	0	1	2	3
Snoring, disturbed sleep	0	1	2	3
Weight loss	0 (nil)	-	2 (yes)	-
Dyspnea at URTI	0	1	2	3
Stridor	0	1 (exertion)	2 (light work)	3 (rest)

**Table 2:** Effect of Kashimas Procedure on phonation

**VHI**	**MPT(Maximum phonation time)**	**GRBAS**
15	17	G1 R1 B1 A0 S0
18	15	G2 R2 B2 A1 S1
27	16	G1 R1 B1 A0 S0
16	14	G2 R2 B1 A2 S2
61	8	G1 R1 B2 A1 S1
24	16	G2 R2 B3 S1 A1
19	14	G2 R1 B2 A2 S1
36	16	G1 R1 B1 A0 S0
38	15	G2 R2 B2 A1 S1
24	15	G2 R2 B1 A2 S2
23	16	G1 R1 B2 A1 S1
13	16	G2 R2 B3 S1 A1

VHI values demonstrated that while nine patients (75%) had no/mild degree of voice handicap, two patients (16.6%) had moderate and one patient (8.33%) had a severe degree of handicap. While 11 patients (91.6%) had a normal MPT (above 10), one patients (8.33%) had a reduced MPT (10 or below). In our study based on dyspnea index score the difference in the preoperative and postoperative mean was 9.3 and the p value was 0.001 which is statistically significant as shown in (Table 3).

**Table 3:** Preoperative and Postoperative mean difference based on dyspnea index score

**Score**	**Prescore**	**Postscore**	**Difference**
Mean	16.1	6.8	9.3
p-value			0.001

**Fig 1:**
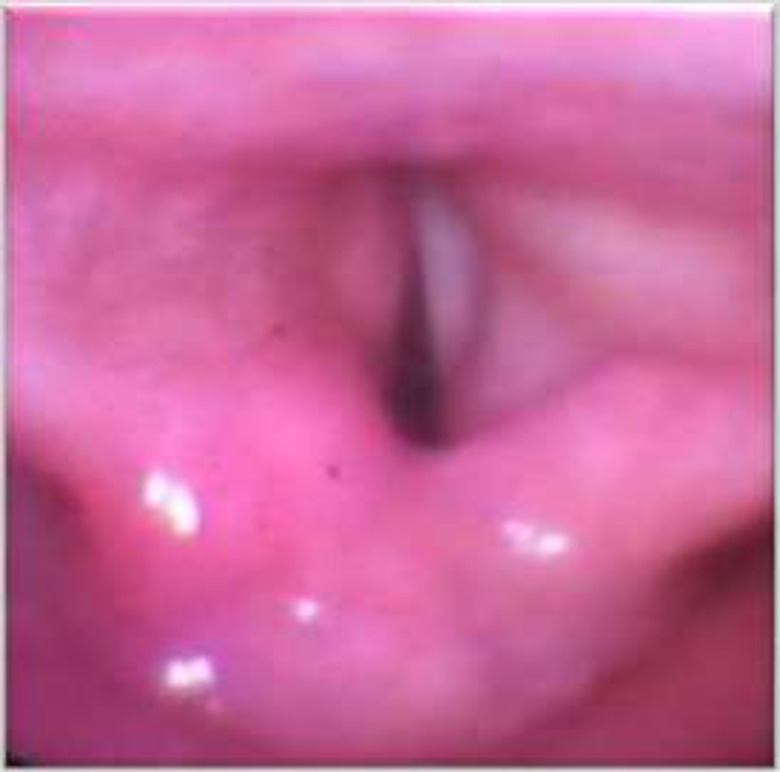
Post-Operative Picture

 According to study by Manju E Issac on effect of Kashimas surgery in bilateral abductor vocal cord palsy which was done on 31 patients the difference in mean preoperative and postoperative scores was 9.29 which is statistically significant and in accordance with our study (17). In our study, none of the patients complained of aspiration. Eckel et al. compared the results between posterior cordotomy and complete arytenoidectomy and found that subclinical aspiration was seen more in patients treated with complete arytenoidectomy (18). Lawson et al. Also in his study he found that arytenoidectomy resulted in subclinical aspiration as compared to posterior cordotomy (19). Figure1 shows the postoperative picture (3 weeks) of Kashimas procedure.

## Conclusion

Kashimas procedure is a satisfactory surgical treatment for treating bilateral vocal cord palsy in regards to strider. No aspiration was seen in any of the patients post-surgery and voice outcome of these patients was also satisfactory with no/ mild voice handicap in 70% of patients and normal MPT in 91.6% of patients.
